# PSMA imaging as a non-invasive tool to monitor inducible gene expression in vivo

**DOI:** 10.1186/s13550-023-01063-5

**Published:** 2024-01-04

**Authors:** Marin Simunic, Jay T. Joshi, Helen Merkens, Nadine Colpo, Hsiou-Ting Kuo, Julian J. Lum, François Bénard

**Affiliations:** 1 Department of Hematology, Clinic for Internal Medicine, Clinical Hospital Centre, Spinciceva 1, 21000 Split, Croatia; 2 Deeley Research Centre, BC Cancer Research Institute, 2410 Lee Avenue, Victoria, BC V8R 6V5 Canada; 3 BC Cancer Research Institute, 675 West 10Th Avenue, Vancouver, BC V5Z 1L3 Canada

## Background

Tetracycline (Tet)-On and Tet-Off systems have been widely used in biomedical research as an important tool allowing for controlled gene expression in eukaryotic cells and organisms [1–4]. Those systems become effective upon binding of a tetracycline class antibiotic [5], either by switching off gene expression in a so-called Tet-Off system or by inducing gene expression in a Tet-On system, wherein a mutant reverse Tet-regulated trans-activator is employed [6, 7]. This non-invasive approach has found wide application in gene therapy and signal cascade activation monitoring, as well as in cell motility tracking studies [8]. Ideally, such system should allow for expression of potentially antigenic proteins in immune competent hosts without triggering an immune response. However, inducible systems require high degrees of biocompatibility and target-to-background contrast and sensitivity [9].

The prostate-specific membrane antigen (PSMA; also known as glutamate-carboxypeptidase II—GCPII; NAALADase; folate hydrolase I—FOLH1) has been widely studied as a drug target for prostate cancer imaging and therapy [9–12]. This enzyme is highly expressed in most metastatic prostate cancers. Favourable results in a phase 3 therapeutic trial, using [^177^Lu]Lu-PSMA-617, were recently reported [13], and several ^18^F- and ^68^ Ga-labelled agents are finding widespread use for diagnostic imaging, with high tumour-to-background ratios allowing for disease detection in most cases of prostate cancer (> 90%) [14–27]. PSMA imaging benefits from enzyme internalization upon binding radiotracers, allowing cellular retention to improve imaging contrast [28–32]. Castanares et al. reported favourable results using PSMA as a reporter gene using adenoviral gene transfer using HCT116 cells, with improved target-to-background ratios over the human sodium iodide transporter and the mutant herpes simplex virus type I thymidine kinase [33].

The aim of this study was to evaluate the feasibility of non-invasive monitoring of inducible gene expression using PSMA as a reporter probe. Such a reporter system could be useful to track inducible gene expression in vivo for research applications. For this purpose, we studied the doxycycline-induced expression of human prostate-specific membrane antigen (hPSMA) in a murine TRAMP-C2 cell line, which does not constitutively express PSMA [34–37].

## Methods

### Cell lines and generation of TRAMP-C2 clones expressing PSMA

TRAMP-C2 cells acquired from American Type Culture Collection (ATCC) and cultured according to ATCC specifications. The TRAMP-C2 cells were transduced with a customized lentiviral vector carrying tetracycline (syn. doxycycline-; “DOX”) inducible PSMA expression system. Creating a DOX-inducible expression system was performed as follows: First, PSMA was sub-cloned out of an expression plasmid EX-G0050-Lv205 (GeneCopoeia, Inc.) with EcoRI and BamH1 and ligated into the target pLVX-TRE3G vector. Next, the ligated product was transformed into E. coli strain DH5alpha for plasmid amplification and verification using restriction digests and gel electrophoresis. To produce lentiviruses, the protocol provided by Clonetech was employed. The lentiviral vector plasmid DNA (PSMA pLVX-TRE3G and pLVX-Tet3G) was diluted with water and added to a tube of Lenti-X Packaging Single Shots provided by Clonetech, and vortexed at high speed. After incubating at room temperature, samples were added dropwise to the 293 T (HEK 293 T) cell culture dishes at 70% confluence. Following 12 h of incubation at 37 °C, 20% O_2_, and 5% CO_2_ in a water-jacketed incubator, fresh complete growth medium was replaced and incubated at 37 °C and 5% CO_2_. At 72 h after the start of transfection, the lentiviral supernatants were harvested and filtered through 0.45-µm filter to remove cellular debris. The filtered pLVX-Tet3G and PSMA pLVX-TRE3G supernatants were stored at -80 ^0^C, thawed slowly on ice, and added to the TRAMP-C2 cells at 70% confluence, at a 1:1 ratio with 4-µg/mL polybrene. The cells were transduced for 12 h at 37 °C and 5% CO_2_ in a water-jacketed incubator, after which the culture medium was discarded and replaced with fresh growth medium.

### *Assessment of *in vitro* induction of PSMA expression by flow cytometry*

The resulting bulk PSMA TRAMP-C2 population was incubated by adding doxycycline at varying concentrations up to 1 µg/ml (2 µM) and incubating for 18 h. For the purpose of flow cytometry analysis, the cells were seeded in V-bottom 96-well plates and pretreated with 1-μg/ml doxycycline hydrochloride (Sigma-Aldrich, St. Louis MO, USA), 18–24 h before the flow cytometry study. Media were removed, and cells were washed with DPBS (Gibco, Carlsbad, CA, USA) supplemented with 2% foetal calf serum and 0.02% NaN_3_, prior to and following a 1-h-long incubation in the dark with 1 ug/ml of Alexa Fluor® 488 anti-human PSMA Antibody (BioLegend, San Diego, CA, USA) per 10^6^ cells. LNCaP cells (maintained in RPMI media supplemented with 10% FBS and 1% penicillin/streptomycin) and wild-type TRAMP-C2 cells were used as positive and negative controls, respectively. Additional negative controls were “unstained” cells—treated with 100–200 μl/well of DPBS with 2% foetal calf serum and 0.02% NaN_3_, and isotype controls, incubated for 1 h with 1 µg/ml Alexa Fluor® 488 Mouse IgG1, κ Isotype Ctrl (FC; Biolegend, San Diego, CA, USA), as well as cells without doxycycline pre-treatment. Flow cytometry runs were performed on q FACScalibur flow cytometer (Becton, Dickinson and Co., Franklin Lake, NJ, USA). Data were analysed using FlowJo software (FlowJo LLC, Ashland, OR, USA).

The “bulk” PSMA TRAMP-C2 cells were then isolated into single-cell clones by limiting dilution in multiple 96-well plates. This method was used to generate a clonal populations, each arising from a single cell. Over the next 2 weeks, these single cells were cultured with G418 (500 µg/mL) and puromycin (3 µg/mL) to isolate clones that have “medium”- and “high”-level PSMA expression. Out of the 25 clones that were tested, four highly expressing populations (designated as clones 1, 14, 16, and 19) were isolated for further experiments. Clones 14 and 19 were validated to be “intermediate” expressors while clones 1 and 16 were high expressors. Protein expression was validated through immunoblotting, using [^177^Lu]Lu-PSMA-617. The four isolated clones were initially maintained in Dulbecco's modified Eagle's medium (DMEM) supplemented with 5% heat-inactivated FBS, 5% Nu-Serum IV, 1% penicillin/streptomycin, 0.005-mg/mL bovine insulin, and 10-nmol/L dehydroisoandrosterone (DHEA) [41], and after 1 week, 300 ug/ml of geneticin (G418) was introduced to the media. Cells were maintained in Dulbecco's modified Eagle's medium (DMEM) supplemented with 10% FBS and 1% penicillin/streptomycin. The cells were confirmed pathogen-free using the IMPACT I PCF profile test (IDEXX BioAnalytics).

### *Assessment of *in vivo* induction of PSMA expression*

All mouse experiments were approved by the Animal Care Committee of the University of British Columbia. 10 × 10^6^ cells of each of the four clones 1, 14, 16, and 19 in 100 μl of media and Matrigel (1:1) were subcutaneously inoculated over the left shoulder of male NOD.Cg-Rag1^tm1Mom^ Il2rg^tm1Wjl/^SzJ (NRG) mice of 12 weeks of age and older (Jackson Laboratory, Bar Harbor, ME, USA), using a 25-gauge needle. The mice were maintained in a pathogen-free animal facility with restricted access on a 12:12 light cycle, monitored for tumour size, weight, and general signs of illness. Five–8 weeks post-inoculation, mice with tumour volume of at least 200 mm^3^ were selected for in vivo imaging and biodistribution studies with the ^18^F-labelled radiotracer DCFPyL [11, 20–22, 42]. A group of mice was pretreated with 50-µg doxycycline per g body weight in 100–200-μl DPBS intraperitoneally, every 24 h for 3 days prior to the study. Additional mice were used as controls and did not receive the antibiotic prior to the study. All animals were randomized to the various groups without considering any other variable than the minimum tumour size. The weight of the mice was 33 ± 3 g at the time of biodistribution. For each clone, a minimum of four mice without administered doxycycline and nine mice with administered doxycycline were used for activity biodistribution in organs. The investigators were not blinded as to which group the animals belonged to during the experimental procedures or data analysis.

For each clone, one mouse was randomly selected for micro-PET-CT imaging before and after doxycycline induction. The mice were intravenously injected with 1.47 ± 0.28 MBq of [^18^F]DCFPyL for biodistribution studies. Ex vivo biodistribution studies were performed immediately post-CO_2_ euthanasia (following anaesthesia in 2% isoflurane in oxygen). Organs were harvested, weighed, and counted on a PerkinElmer WIZARD 2480 gamma counter (PerkinElmer Inc., Waltham, MA, USA). Organ uptake was calculated in per cent injected activity per gram of tissue (%ID/g), and an unpaired Student’s t-test was performed using GraphPad Prism 8 (GraphPad Software Inc., San Diego, CA, USA) with tumour uptake post-doxycycline induction in the test group and tumour uptake without doxycycline induction in the control group. The cutoff for significance was a *p*-value under 0.05.

For imaging, at least one mouse from each group was randomly selected to be used before and after tetracycline induction so that the animal served as its own control. For both imaging sessions, the mice received and 5 ± 0.86 MBq of [^18^F]DCFPyL. Following baseline imaging in the absence of tetracycline, the mice recovered from anaesthesia and were treated with 50-µg doxycycline per g body weight in 100–200-μl dPBS intraperitoneally, every 25 h for 3 days prior to the subsequent study.

PET-CT images were acquired one hour post-tracer injection using Siemens Inveon micro-PET-CT scanner (Siemens Medical Solutions, Knoxville, TN, USA) and analysed using the Inveon Research Workplace (Siemens Medical Solutions, Ann Arbor, MI, USA). PET-CT images were compared side by side for the same mouse before and after induction using the same uptake bar with colour spectrum corresponding to the percentage of injected activity per gram tissue.

## Results

### Detectable hPSMA in TRAMP-C2 cell lines

**Fig. 1:**
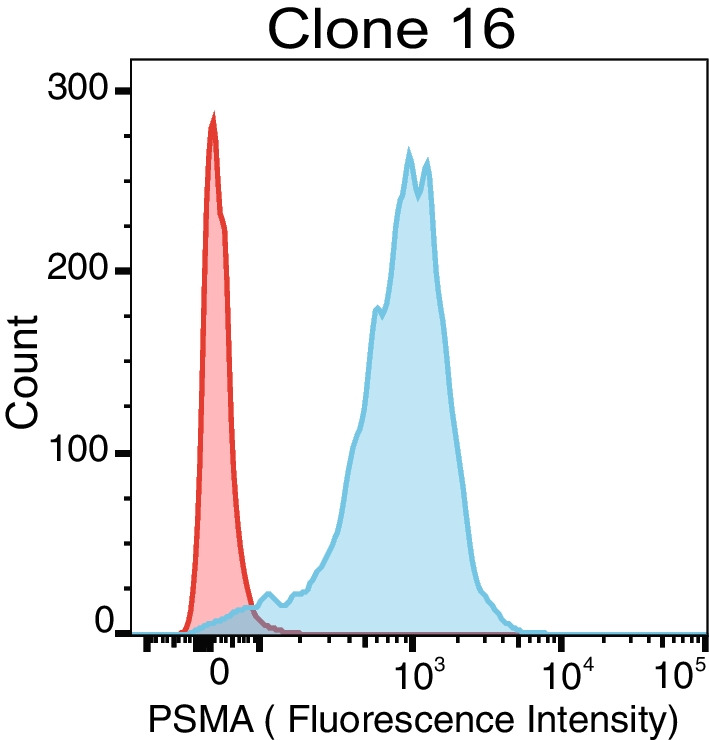
Flow cytometry results of PSMA expression. PSMA-expressing clones were isolated from the “bulk” population. The histogram shows a representative clone (clone 16) with high expression under doxycycline induction (blue) compared to non-induced control (red)

The results of in vitro flow cytometry of a representative example of a parental clone are shown in Fig. 1 as histogram plots of cell count versus FITC fluorescence. The results of flow cytometry from the four clones are available in Additional file 1: Fig. S1. Except for the LNCaP cells as positive control, doxycycline-untreated cells showed identical negative PSMA-expressing profiles for unstained, isotype-stained, and anti-PSMA-stained tests. Upon doxycycline induction, all four clones showed increase in PSMA expression upon doxycycline induction with a shift in mean channel fluorescence signal for anti-PSMA-stained populations, except the negative control (wild-type TRAMP-C2 cells).

### In vivo* PSMA expression in immunocompromised mice—radiopharmaceutical biodistribution and PET-CT imaging studies*

**Fig. 2:**
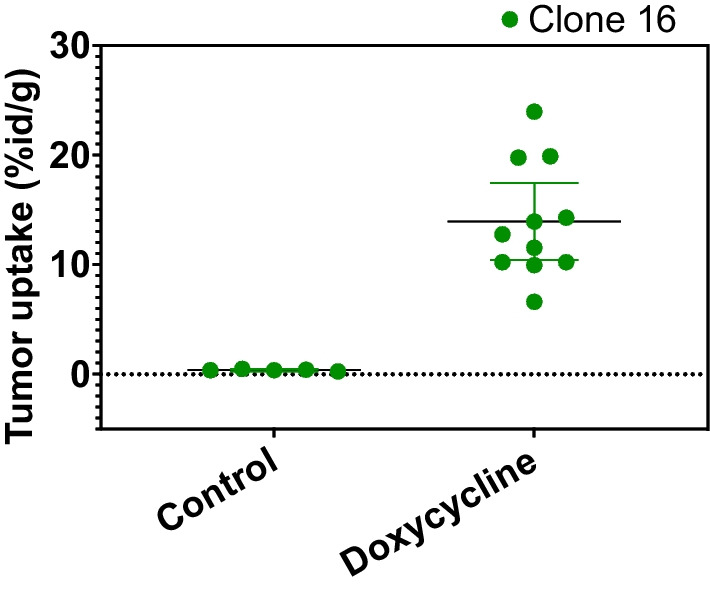
In vivo PSMA expression levels in different clones before and after doxycycline induction. Transfected TRAMP-C2 clones express PSMA upon doxycycline induction in vivo*.* Unpaired *t*-tests for all four clones showed a *p*-value of < 0.0001 when comparing radioactivity uptake of [^18^F]DCFPyL in mice with or without pre-treatment with doxycycline. The figure shows the %ID/g of tumour uptake in the induced (doxycycline) mice compared to the control group for clone 16

**Fig. 3:**
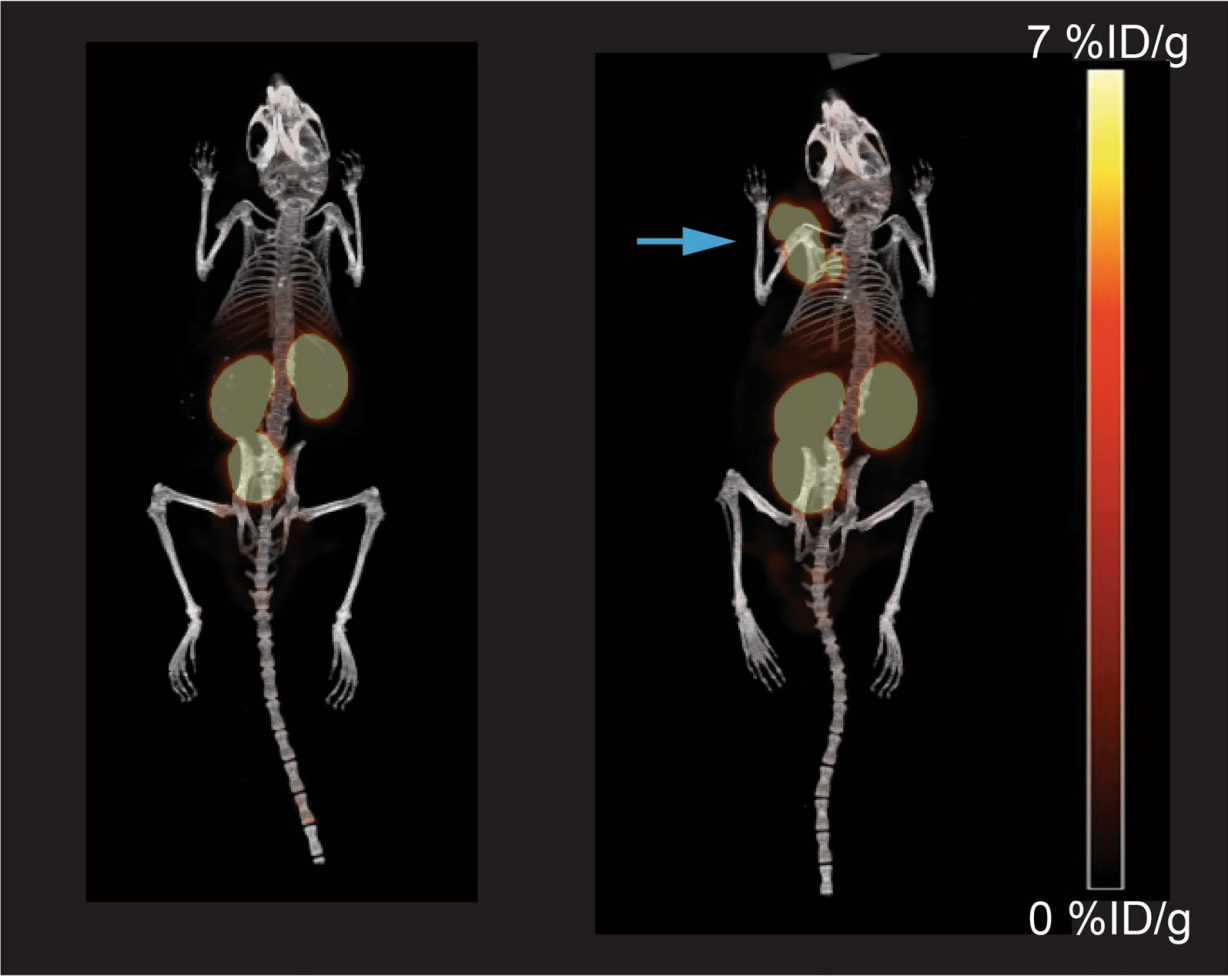
Comparison of PET-CT images before and after doxycycline induction. Comparison of PET-CT images before (left) and after (right) doxycycline induction in a representative mouse (clone 16). Uptake in the tumours was only visualized after doxycycline administration. The spectrum bar has a range 0–7.1%ID/g for PET (yellow/red tones). The tumour is indicated with a blue arrow

Uptake of [^18^F]DCFPyL in NRG mice followed a similar pattern of distribution in different organs as in studies using LNCaP cells [43], with higher degrees of variability in uptake in adrenal (2.17 ± 2.3% ID/g) and seminal glands (5.04 ± 12.32% ID/g) due to possible urine contamination of these organs during harvesting. Pre-treatment with doxycycline significantly impacted the level of [^18^F]DCFPyL uptake in tumours (Fig. 2). Clone 19 showed significantly lower uptake (Additional file 1: Fig. S2). An unpaired t-test resulted in *p*-values under 0.0001 for all four clones, when comparing uptake levels without induction versus post-induction. PET-CT images of four mice, each inoculated with a different clone, confirmed this finding when images before and after induction were compared. In addition, tumours could only be visualized with high tumour-to-background ratio after undergoing induction with intraperitoneal doxycycline injection (Fig. 3 and Additional file 1: Fig. S3).

## Discussion

Both in vitro and in vivo*,* our experiments confirmed viability of the cells in vivo and the inducibility of PSMA expression in all four transduced TRAMP-C2 clones.

A limitation of our study was that we did not perform a comprehensive evaluation of the kinetics of protein induction which could provide useful information on the duration of protein induction and rate of resolution [7]. We did not perform further isolation of high PSMA-expressing uniform clonal populations, marked by reliably uniform protein expression and uptake patterns that could be used for therapy studies. Finally, blinding the investigators as to which group the animals belonged to would have further improved the study design by avoiding selection bias.

No adverse reactions were seen with the tumour model, and the uptake in other organs followed a similar pattern as LNCaP-bearing mice. This confirms the potential of PSMA as a reporter gene to monitor successful gene transfer and induction in vivo. Several new treatment strategies that rely on direct, viral, or cell-based gene therapies are being used in clinical practice or investigated in clinical trials [49, 50]. From CAR-T cells, mRNA vaccines, oncolytic viruses, and various viral vectors, the ability to monitor in vivo, non-invasively, gene expression in vivo, either in preclinical models or clinical trials, can be useful to improve the design of gene transfer methods and protocols. For example, studying kinetics can provide information about viability and proliferation of the engineered cells [51]. In addition, tracers targeting PSMA, such as [^18^F]DCFPyL used in our studies, are becoming widely available, improving the feasibility of reporter gene monitoring for clinical research studies. In our case, we developed this approach as part of establishing an immunocompetent tumour model that can expresses PSMA expression in vivo*.* As an initiate step, this study serves as proof-of-concept that PSMA imaging can be used as a reported gene to monitor inducible gene expression in vivo*.*

## Conclusion

Our aim was to demonstrate the feasibility to use non-invasive imaging to monitor doxycycline-dependent PSMA expression in vivo*.* Flow cytometry studies confirmed that all the parental clones provided to us were suitable for proceeding with in animal experiments. Proof of induction in vivo was confirmed by ex vivo biodistribution data and in vivo imaging. Collectively our data show that there is a significant increase in the uptake of radiotracers post-PSMA induction confirming the application potential of this model as an effective reporter for tetracycline-induced gene expression in vivo.

### Supplementary Information

**Additional file 1.** **Supplemental Figure 1.** Flow cytometry results of PSMA expression in clones 1, 14, 16 and 19. **Supplemental Figure 2.** In vivo PSMA-expression levels in different clones before and after doxycycline induction. **Supplemental Figure 3.** Comparison of PET-CT images before and after doxycycline induction.

## Data Availability

The datasets used and/or analysed during the current study are available from the corresponding author on reasonable request.

## Abbreviations

% ID/g: Per cent injected activity per gram
[^177^Lu]Lu-PSMA617: Lutetium-177-labelled vipivotide tetraxetane–a lutetium-177-labelled PSMA-targeting radioligand
18F: Fluorine-18
[^18^F]DCFPyL: 2-(3-{1-Carboxy-5-[(6-[^18^F]fluoro-pyridine-3-carbonyl)-amino]-pentyl}ureido)-pentanedioic acid
^68^Ga: Gallium-68
C57Bl/6: Most widely used laboratory mouse strain
CAR-T: Engineered chimeric antigen receptor-carrying T-cells
CT: Computed tomography
DCFPyL: See [18F]DCFPyL
DHEA: Dehydroisoandrosterone
DMEM: Dulbecco’s modified Eagle’s medium
DPBS: Dulbecco's phosphate-buffered saline
et al.: And others (Latin et alii/aliae)
FBS: Foetal bovine serum
FITC: Fluorescein isothiocyanate
FL1-H: Height in the FL1 channel
G418: Geneticin, a selection antibiotic
GCPII: Glutamate carboxypeptidase II (GCPII), also known as N-acetyl-L-aspartyl-L-glutamate peptidase I (NAALADase I), NAAG peptidase, or prostate-specific membrane antigen (PSMA)
hPSMA: Human PSMA
IgG1: Subclass of immunoglobulin G
IMPACT: PCR-based pathogen testing
LLC: Limited liability company
LNCaP: Human metastatic prostate cancer cell line (originated from a lymph node metastasis cancer of the prostate)
MBq: Megabecquerel
mRNA: Messenger ribonucleic acid
NAAG: N-acetyl-L-aspartyl-L-glutamate peptidase I, NAAG peptidase, GCPII, PSMA
NAALADase: I N-acetyl-L-aspartyl-L-glutamate peptidase I (NAALADase I), NAAG peptidase, GCPII, PSMA
Nu-serum™: A growth medium supplement
NRG: NOD.Cg-Rag1tm1Mom Il2rgtm1Wjl/SzJ (“NRG”, NOD-Rag1null IL2rg null, NOD rag gamma) mice–an immunodeficient mouse strain
PET: Positron emission tomography
PSMA: Prostate-specific membrane antigen
PSMA617: (Also PSMA-DKFZ-617) vipivotide tetraxetan
RPMI-1640: Roswell Park Memorial Institute 1640—culture medium
SV40: Simian virus 40
T-cell: Lymphocyte(s) primed in the thymus gland, expressing T-cell receptor (CD3+)
tet: Tetracycline
Tet-On: Tetracycline-controlled transcriptional activation in the presence of tetracycline or its derivatives
Tet-Off: Tetracycline-controlled transcriptional activation in the absence of tetracycline or its derivatives
TRAMP: Murine prostate cancer cell line (transgenic adenocarcinoma of mouse prostate)
TRAMP-C2: Murine prostate cancer cell line mimicking intermediate metastatic progression
°C: Degree Celsius
