# Supplementary material for: PSMA imaging as a non-invasive tool to monitor inducible gene expression in vivo
Source: EJNMMI Res. 2024 Jan 4;14:3. doi: 10.1186/s13550-023-01063-5 (PMC10767034; doi:10.1186/s13550-023-01063-5)
Supplement: Supplementary file 1 — Additional file 1. Supplemental Figure 1. Flow cytometry results of PSMA expression in clones 1, 14, 16 and 19. Supplemental Figure 2. In vivo PSMA-expression levels in different clones before and after doxycycline induction. Supplemental Figure 3. Comparison of PET-CT images before and after doxycycline induction. [file 13550_2023_1063_MOESM1_ESM.docx]

**PSMA expression as a non-invasive tool to monitor gene expression in an inducible tumour model.**

Simunic M, Joshi J, Merkens H, Colpo N, Kuo HT, Lum JJ, Bénard F.

**Supplemental Data Section**

**Supplemental** **Figure 1. Flow cytometry results of PSMA expression in clones 1, 14, 16 and 19.** Four PSMA expressing clones were isolated from the “bulk” population. Transduced TRAMP-C2 cells were single cell sorted to acquire clonal populations of PSMA expressing cells. Four clonal populations were isolated after cell sorting. (A) Histograms and (B) corresponding bar graph showing that clones 1 and 16 were high-PSMA expressing clones while clones 14 and 19 were medium expressing clones as analyzed through flow cytometry. Orange: doxycycline-uninduced cells; blue: isotype controls; red: doxycycline-induced cells. Error bars + SEM.

**Supplemental Figure 2.** ***In vivo* PSMA-expression levels in different clones before and after doxycycline induction.** Transfected TRAMP-C2 clones express PSMA upon doxycycline-induction *in vivo.* Unpaired t-tests for all four clones showed a p-value of <0.0001 when comparing radioactivity uptake of [^18^F]DCFPyL in mice with or without pre-treatment with doxycycline. Significantly lower (p=0.0001, unpaired t-test) uptake was seen with clone 19 (mean uptake = 6.96% ID/g) when compared to clones 1, 14 and 16 combined (mean uptake = 15.43 % ID/g).


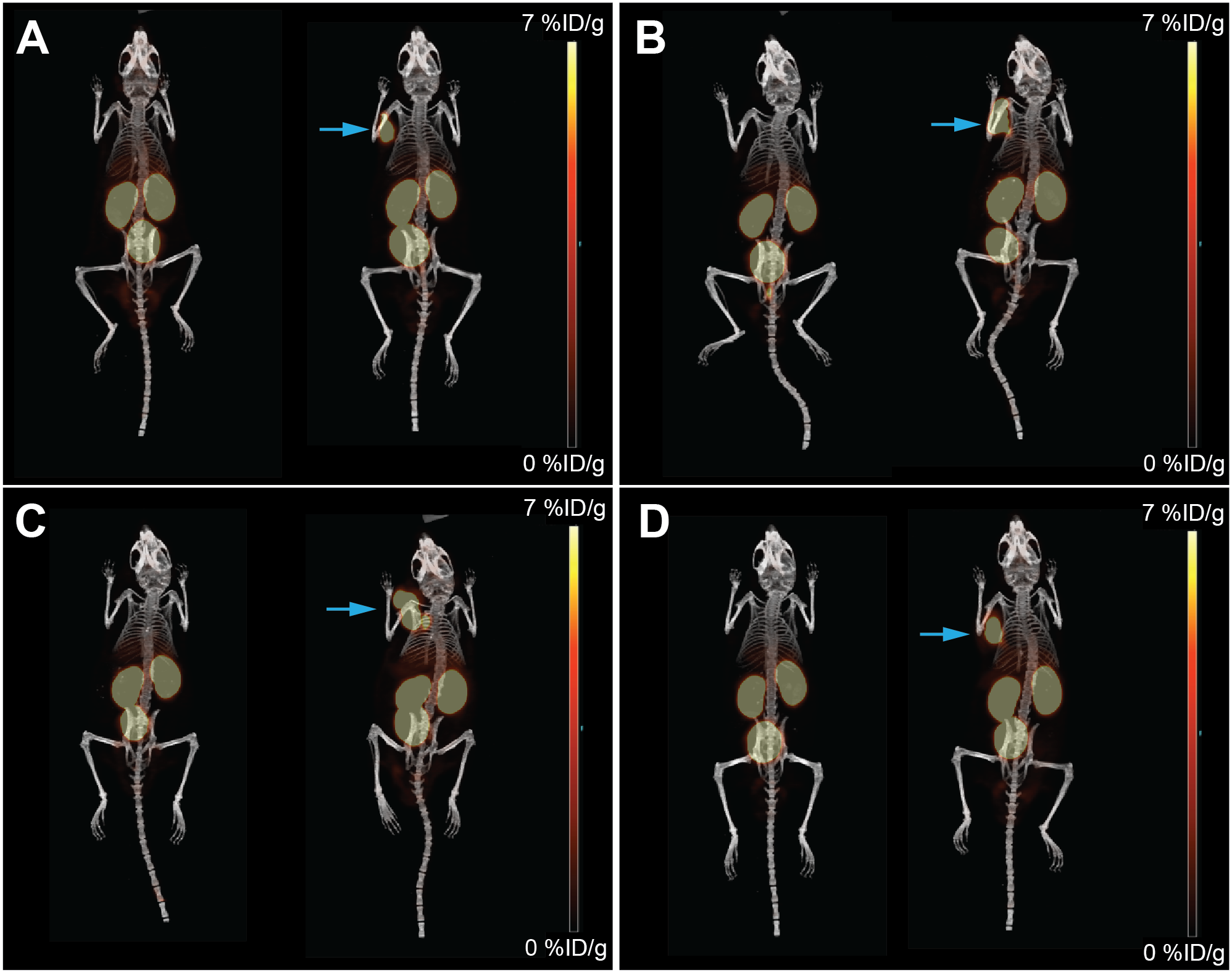


**Supplemental Figure 3. Comparison of PET-CT images before and after doxycycline induction.** For each panel A-D, comparison of PET-CT images before (left) and after (right) doxycycline-induction in 4 mice, each inoculated with one of the four transfected TRAMP-C2 clones: 1 (A), 14 (B), 16 (C) and 19 (D). All mice received intravenously 5 ± 0.86 MBq of [^18^F]DCFPyL one hour before imaging. Uptake in the region tumours were inoculated was only visualized after doxycycline administration. Physiological uptake was visualized in urinary bladder, kidneys, salivary glands, and, to a lesser extent, in liver and pancreas. Corresponding to all images are spectrum bars of 0-7.1 %ID/g for PET (yellow/red tones). Tumours (blue arrow).
